# Treatment of Ethmoidal Dural Arteriovenous Fistulae Using Supraorbital Keyhole Subfrontal Approach

**DOI:** 10.3390/medicina60071128

**Published:** 2024-07-12

**Authors:** Tsung-Hao Li, Chun-Ting Chen, Yuan-Yun Tseng, Ching-Chang Chen, Tao-Chieh Yang

**Affiliations:** 1 School of Medicine, Chung Shan Medical University, Taichung City 402, Taiwan, China; alimama000@gmail.com; 2 Department of Neurosurgery, Linkou Chang Gung Memorial Hospital, Taoyuan City 33305, Taiwan, China; b9002055@cgmh.org.tw (C.-T.C.); jcchen130@gmail.com (C.-C.C.); 3 Department of Neurosurgery, New Taipei Municipal Tu-Cheng Hospital (Built and Operated by Chang Gung Medical Foundation), New Taipei City 236, Taiwan, China; britsey@tmu.edu.tw; 4 Department of Neurosurgery, Chung Shan Medical University Hospital, Taichung City 402, Taiwan, China

**Keywords:** ethmoidal dural arteriovenous fistula (eDAVF), supraorbital keyhole surgery, neuronavigation, intracranial vasculature

## Abstract

Due to a unique cortical venous drainage pattern without sinus drainage, ethmoidal dural arteriovenous fistula (DAVF) are uncommon cerebral vascular lesions that carry a high risk of brain bleeding and neurologic deficit. Surgical intervention has been found to have a lower complication rate and a more satisfactory obliteration rate than endovascular treatment among the various DAVF treatment options. The supraorbital keyhole subfrontal approach is one of the least invasive and appropriate surgical techniques for addressing the anterior fossa vascular lesion in eDAVFs. We describe two men, ages 60 and 71, who underwent this surgical intervention to treat asymptomatic Cognard type IV eDAVFs. Complete obliteration with a detached fistulous point and skeletonization was accomplished with the aid of intraoperative neuronavigation. Thus, we suggest that a suitable surgical method for the treatment of eDAVFs would be to use a supraorbital keyhole subfrontal approach.

## 1. Introduction

Anomalous arteriovenous shunts between meningeal arteries and dural venous sinuses or cortical veins are known as dural arteriovenous fistulas (DAVFs). This abnormality accounts for 10–15% of all intracranial arteriovenous malformations [1]. Ethmoid branches of the ophthalmic artery provide eDAVFs, which make up about 2–3% of DAVFs [1]. eDAVFs often drain into the dilated cortical vein without sinus drainage [2,3]. Some very dangerous venous drainage features, such as direct cortical venous drainage, cortical vein reflux (CVR), and venous ectasia, are associated with a high risk of cerebral bleeding and severe neurological impairments. These traits frequently indicate a malignant nature proclivity in eDAVFs [4,5]. Most eDAVFs with CVR necessitate prompt treatment [2,3,4]. For eDAVFs, endovascular embolization and surgery are the two major treatments. Surgery, in particular, offers a significantly lower risk of complications than endovascular techniques, as well as an outstanding record of completely obliterating eDAVFs [3,6,7]. Bifrontal interhemispheric, low subfrontal, pterional, unilateral high frontal, and transfrontal sinus approaches are among the several surgical approaches for eDAVFs [3,7,8,9]. In this case report, we describe successful mini-invasive surgery for treating Cognard type IV eDAVFs in two cases using a unilateral supraorbital keyhole subfrontal approach.

## 2. Case Report

**Figure 1:**
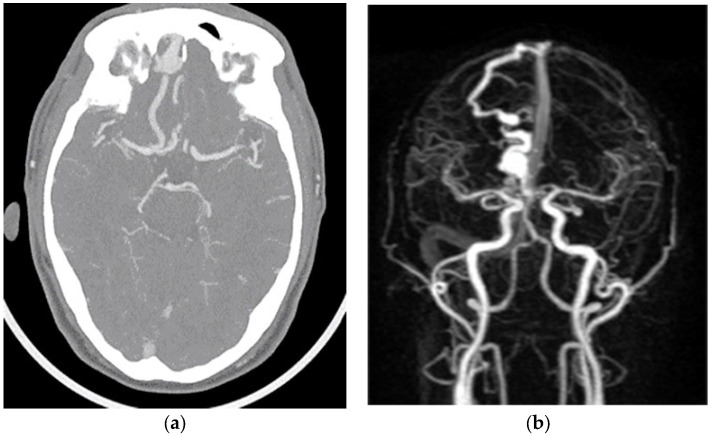
(**a**) Preoperative computer tomography scan of head and neck; (**b**) preoperative brain magnetic resonance angiography.

**Figure 2:**
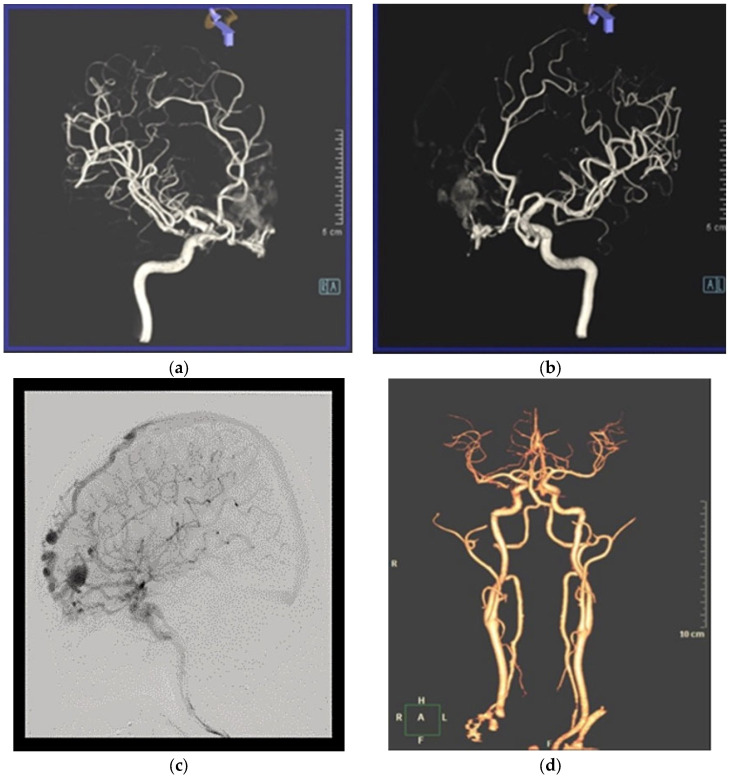
(**a**) Preoperative right oblique view of cerebral angiography; (**b**) preoperative left oblique view of cerebral angiography; and (**c**) preoperative cerebral angiography; (**d**) brain–computer tomography angiography for postoperative follow-up.

**Figure 3:**
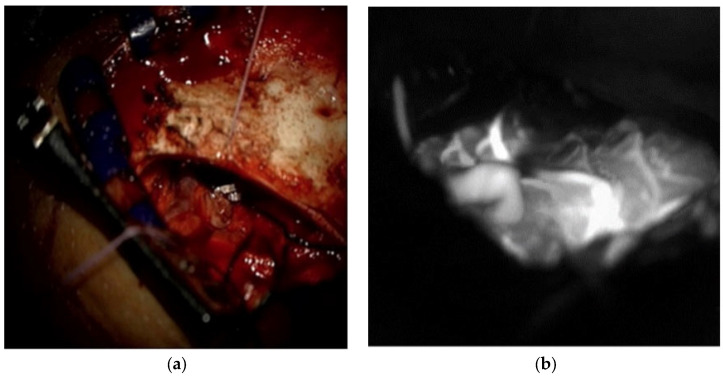
(**a**) Intraoperative image of eDAVF clamped with 2 titanium clips and remission of arterialized vein; (**b**) intraoperative indocyanine green image.

Case 1:

In December 2022, a 60-year-old man with a high fever and Lemierre syndrome diagnosis was admitted to Chung Shan University Hospital. After the patient’s admission, a head and neck computer tomography (CT) scan (Figure 1a) and a brain magnetic resonance angiography (Figure 1b) were performed to assess the cause of the fever. These tests unintentionally revealed a cerebral vascular lesion in the right anterior frontal base area without any bleeding or infarction. A right anterior frontal vein with a large venous ectasia that drained back to the superior sagittal sinus (SSS) and a right frontal basal vein that drained back to the inferior petrosal sinus were the two cortical veins with CVR that were fed by the ethmoidal branches of the bilateral ophthalmic arteries, according to brain digital subtraction angiography (DSA) (Figure 2a–c). The patient had neither a neurologic deficiency nor any symptoms. Cognard type IV was assigned to the eDAVF (Figure 2c). We scheduled a surgical procedure for eDAVF treatment a month after the Lemierre syndrome patient finished receiving antibiotic therapy. First, we used neuronavigational guidance (CranialMap 3.0 Navigation Software, Stryker NAV3i Platform). The skin incision was made from the right supraorbital incisura in the eyebrow, starting laterally and moving medially. Next, a high-speed electric burr and saws were used to accomplish a right supraorbital keyhole craniotomy. After releasing the cerebrospinal fluid, the surgeon detached the bilateral cribriform galli fistulous site and used micro scissors and bipolar coagulation to skeletonize the right frontal basal vein. Two titanium clips clamped the right anterior frontal vein at the location of the DAVF frontal base dura fistula point. There was no disruption whatsoever to the olfactory bulbs and tracts. At the frontal lobe section, the right anterior cortical veins were unharmed (Figure 3a). We performed intraoperative indocyanine green (ICG) angiography to verify the absence of early arterialized venous outflow into the right anterior frontal vein (Figure 3b). A follow-up brain CT angiography performed one month after surgery revealed that the prior eDAVF had been effectively obliterated (Figure 2d). After an uncomplicated postoperative stay, the hospital discharged the patient one week after the procedure. He was then routinely monitored in the outpatient department for six months, during which he did not exhibit any new neurologic deficits or symptoms except for numbness in the right supraorbital craniotomy region.

CASE 2:

In April 2024, a 71-year-old man was found with a right side eDAVF incidentally in Linkou Chang Gung Memorial Hospital. An initial brain CTA showed a right frontal base vascular lesion. The right superficial temporal artery and the right ethmoidal branches of the right ophthalmic artery feeded blood into the eDAVF. The vein located at the right frontal base was twisted and engorged, with a venous ectasia, and served as the drainage vein of the eDAVF. The eDAVF was designated as a Cognard type IV. We adapted the right supraorbital keyhole craniotomy for the asymptomatic eDAVF. The brain CT angiography, cerebral angiography, operation procedure, and intraoperative can be seen in the video 1 (https://youtu.be/la28gyGxIek?si=q7MgPs37eyjQGW8B, accessed on 31 May 2024).

The patient had an uneventful clinical course after surgery.

## 3. Discussion

Today, there are three different modalities to treat DAVFs such as transcatheter embolization, surgery, stereotactic radiosurgery, or combination treatment [2,7,10,11]. Since endovascular embolization is low invasive and has a good effect, it has long been thought of as the first-line treatment for DAVFs [2,11]. In certain types of DAVFs, radiosurgery seems to be effective when endovascular or surgical therapy is unable to completely eradicate the residual nidus [10,11]. Intracranial DAVFs affecting convexity, foramen magnum, craniocervical junction, and eDAVFs are amenable to surgical treatment [7,11]. When determining the risk associated with each dural AVF, the Cognard classification offers valuable information on shunt placement, venous drainage characteristics, and venous outflow angioarchitecture [12]. This information facilitates decision-making on the most suitable course of treatment. 

eDAVFs, which account for 2–3% of intracranial DAVFs [1], are notorious for their tendency to drain directly into cortical veins with or without venous ectasia [4,13]. The incidence of bleeding was sevenfold higher in Cognard type IV DAVF patients (3.5% without ectasia vs. 27% with ectasia), which is defined by venous draining into the cerebral vein with venous ectasia [4]. Clinical signs and symptoms of eDAVFs in the anterior ethmoidal area include headache, tinnitus, exophthalmos, and intracranial hemorrhage (ICH). The natural course of eDAVFs is usually aggressive and carries a high risk of ICH because of the unique way veins drain in this area. This is why early treatments are often needed [4,6,11]. The treatment of eDAVFs using several modalities has been verified in the literature [6,7,14]. Surgical disconnection is still the most effective way to treat eDAVFs. Vision loss resulting from ophthalmic artery injury, particularly to the central retinal artery during catheterization, is a serious concern when validating endovascular embolization to treat eDAVFs [3,6,7,14]. Moreover, endovascular techniques, whether transarterial or transvenous, faced significant challenges due to venous ectasia, retrograde cortical vein reflux, and direct draining into the somewhat tortuous cortical veins [6,14]. In contrast to earlier studies that used endovascular methods, surgical therapy for eDAVFs typically achieved greater complete obliteration and a lower complication rate by disconnecting the cortical draining vein from the fistula locations using clips or coagulation [6,7]. We underwent surgical therapy even though the asymptomatic cases of Cognard type IV to prevent significant risk of ICH.

Surgical approaches are versatile, including frontal, bifrontal, pterional, transfrontal sinus, or unilateral orbitozygomatic craniotomy [3,8,15,16]. A supraorbital keyhole subfrontal approach through an eyebrow skin incision was used to minimize surgical-related morbidity due to smaller wound exposure and smaller craniotomy size than the traditional surgical approach [17,18]. The supraorbital keyhole approach was a paramedian craniotomy, not crossing the midline, which reduced the subsequent risk of damage to the dura and the underlying veins, including those enlarged by the fistula. By contrast, such a maneuver as bifrontal craniotomy, when in close proximity to or crossing the midline, may injure the SSS or DAVF arterialized veins [19]. Intraoperative neuronavigational guidance was performed to define the optimal surgical trajectory. The incision can also be at the location of the eyebrows, which has a better cosmetic effect than other surgical techniques [9,19]. The target eDAVF was clearly shown in the surgical field through the supraorbital keyhole subfrontal approach, and the elimination of the eDAVF was also successfully achieved by disconnecting the cortical draining vein from the fistula points by clips or coagulation without perioperative complications [17].

The disadvantages of the supraorbital keyhole approach were illumination and a narrow viewing angle that may require frequent adjustments of the operating table and microscope for adequate visualization of a given lesion. Endoscopes could produce illumination at depth rather than from a distance, allowing them to illuminate the area of interest without casting shadows on the field [18,19]. Avoidance of the supraorbital nerve injury and breach of the frontal sinus intraoperatively would limit surgical exposure of the sagittal midline or contralateral frontal base area. Staying at least 5 mm lateral to the supraorbital notch or foramen with the craniotomy significantly reduced the risk of supraorbital palsy. Neuronavigation can help the surgeon to accurately locate and target the lesion, improving the precision of the procedure [18,19]. In our opinion, for asymptomatic patients with eDAVF, Cognard type IV, the supraorbital keyhole subfrontal approach should be considered as the first-line treatment to eliminate the risk of possible malignant clinical course.

## 4. Conclusions

We recommend early treatment for asymptomatic individuals with eDAVFs who have direct cerebral vein drainage with or without venous ectasia. This is to prevent the possibility of a malignant clinical progression. The supraorbital keyhole subfrontal technique, in combination with neuronavigation, offers a minimally invasive yet efficient approach to treat eDAVFs if patients are meticulously chosen.

## Data Availability

The clinical data (patient data, pictures, etc.) were all retrieved with the consent of the patients and the hospital involved. All referential data were retrieved with consent.

## Abbreviations

eDAVF	ethmoidal dural arteriovenous fistula
CVR	cortical vein reflux
CT	computer tomography
DSA	digital subtraction angiography
ICG	indocyanine green
SSS	superior sagittal sinus
